# Extracellular matrix with defective collagen cross-linking affects the differentiation of bone cells

**DOI:** 10.1371/journal.pone.0204306

**Published:** 2018-09-25

**Authors:** Takako Ida, Masaru Kaku, Megumi Kitami, Masahiko Terajima, Juan Marcelo Rosales Rocabado, Yosuke Akiba, Masako Nagasawa, Mitsuo Yamauchi, Katsumi Uoshima

**Affiliations:** 1 Division of Bio-Prosthodontics, Niigata University Graduate School of Medical and Dental Sciences, Niigata, Japan; 2 North Carolina Oral Health Institute, University of North Carolina at Chapel Hill, Chapel Hill, NC, United States of America; University of Oulu, FINLAND

## Abstract

Fibrillar type I collagen, the predominant organic component in bone, is stabilized by lysyl oxidase (LOX)-initiated covalent intermolecular cross-linking, an important determinant of bone quality. However, the impact of collagen cross-linking on the activity of bone cells and subsequent tissue remodeling is not well understood. In this study, we investigated the effect of collagen cross-linking on bone cellular activities employing a loss-of-function approach, using a potent LOX inhibitor, β-aminopropionitrile (BAPN). Osteoblastic cells (MC3T3-E1) were cultured for 2 weeks in the presence of 0–2 mM BAPN to obtain low cross-linked collagen matrices. The addition of BAPN to the cultures diminished collagen cross-links in a dose-dependent manner and, at 1 mM level, none of the major cross-links were detected without affecting collagen production. After the removal of cellular components from these cultures, MC3T3-E1, osteoclasts (RAW264.7), or mouse primary bone marrow-derived stromal cells (BMSCs) were seeded. MC3T3-E1 cells grown on low cross-link matrices showed increased alkaline phosphatase (ALP) activity. The number of multinucleate tartrate-resistant acid phosphatase (TRAP)-positive cells increased in RAW264.7 cells. Initial adhesion, proliferation, and ALP activity of BMSCs also increased. In the animal experiments, 4-week-old C57BL/6 mice were fed with BAPN-containing diet for 8 weeks. At this point, biochemical analysis of bone demonstrated that collagen cross-links decreased without affecting collagen content. Then, the diet was changed to a control diet to minimize the direct effect of BAPN. At 2 and 4 weeks after the change, histological samples were prepared. Histological examination of femur samples at 4 weeks showed a significant increase in the number of bone surface osteoblasts, while the bone volume and surface osteoclast numbers were not significantly affected. These results clearly demonstrated that the extent of collagen cross-linking of bone matrix affected the differentiation of bone cells, underscoring the importance of collagen cross-linking in the regulation of cell behaviors and tissue remodeling in bone. Characterization of collagen cross-linking in bone may be beneficial to obtain insight into not only bone mechanical property, but also bone cellular activities.

## Introduction

Bone is a dynamic mineralized tissue composed of organic extracellular matrix (ECM) and inorganic minerals, supporting the body frameworks and providing mineral homeostasis of body fluids. Type I collagen is the most abundant ECM component in bone, comprising approximately 90% of total proteins. Biosynthesis of type I collagen is a long and complex process, which includes a series of post-translational modifications [1]. Intra- and extra-cellular post-translational modifications of specific lysine residues are crucial for the formation of covalent collagen cross-links, fibrillogenesis and the stability of fibrils [2]. The amount, type, and maturation of collagen cross-links vary from tissue to tissue, and these differences are most likely related to the physiological functions of different tissues [3]. Notably, the type and composition of collagen cross-links in bone vary with age, pathological condition, loading status, bone type, and anatomical locations [4–6]. This also contributes to the wide spectrum mechanical properties of bone [7, 8].

Biochemical and biophysical properties of type I collagen are known to affect cell behaviors, including survival, proliferation, and differentiation [9–12]. For instance, cellular response to monomeric or denatured collagen differs from the response to the naturally formed collagen fibrils [13–17]. Fibroblasts on fibrillar collagen gels have a decreased spreading and actin cytoskeleton organization compared to that of cells cultured on monomeric collagen [13]. Compared to polymerized type I collagen, monomeric collagen stimulates the proliferation of arterial smooth muscle [14], mesangial [18], and melanoma [15] cells. Additionally, matrix elasticity affects the osteoblastic differentiation of osteoblasts [19] and mesenchymal stem cells (MSCs) [20]. As collagen cross-linking contributes to fibrillogenesis, matrix stability and elasticity [3, 21, 22], it is possible that the changes in collagen cross-linking affect the activity of cells and subsequent tissue remodeling in bones. Therefore, we hypothesized that an extent of collagen cross-linking affects bone cellular activities.

The bone remodeling involves bone-resorption by osteoclasts and bone formation by osteoblasts. The highly coordinated balance between osteoblasts- and osteoclasts-activity is key to the maintenance of bone volume. Therefore, their reciprocal actions are important for the bone homeostasis. Although the effect of collagen cross-linking on osteoblast activity was partially elucidated, the results are still not conclusive, because most of the previous studies investigated the differentiation of osteoblasts in the presence of a cross-linking inhibitor [23–25]. Under this condition, it is not clear if the effect on cells was due to altered collagen cross-linking or the action of the cross-linking inhibitor on the cells. Even though some data were acquired under the BAPN-free condition, the effect of low-cross-linked matrices on osteoblasts has not been conclusive [23]. Thus, to obtain further insight into this, we established an experimental model that permitted an assessment of the effect of collagen cross-linking on various cell types, including osteoclasts, under the cross-link inhibitor-free condition *in vitro* and *in vivo*.

## Materials and methods

### Cell culture and matrix preparation

A mouse calvarial pre-osteoblastic cell line, MC3T3-E1 (MC) subclone 4, was obtained from the American Type Culture Collection (ATCC) (Manassas, VA, USA) and maintained in alpha-minimum essential medium (alpha-MEM) (Life Technologies, Carlsbad, CA, USA) supplemented with 10% fetal bovine serum (FBS) and 1% penicillin/streptomycin at 37°C in a humidified atmosphere with 5% CO_2_. The culture medium was replaced twice a week. MC cells were seeded onto 35-mm dishes at a density of 2 × 10^5^ cells/dish. After 2 days, the medium was replaced with the matrix formation medium (alpha-MEM supplemented with 10% FBS, 1% penicillin/streptomycin, and 50 μg/mL of ascorbic acid (Sigma-Aldrich, St. Louis, MO, USA)), with or without β-aminopropionitrile (BAPN) (0–2 mM; Tokyo Chemical Ind., Co., Ltd, Tokyo, Japan), and cultured for 2 weeks. The cell-matrix layers were washed with ice cold phosphate buffered saline (PBS) three times and stored at -20°C. The matrices were processed for biochemical/histological analyses or used for cell culture substrates as outlined below.

### Type I collagen component assay by SDS-PAGE

Cultured matrices were directly dissolved in sodium dodecyl sulfate (SDS) sample buffer (Life Technologies), heated at 80°C for 10 min, and centrifuged at 8,000 *× g* for 20 min. Equal volumes of supernatant were loaded onto the NuPAGE 3–8% Tris-Acetate gel (Life Technologies), and electrophoresis was performed. Gels were directly stained with Coomassie Brilliant Blue R-250 (CBB) (Wako Pure Chemicals Ind., Ltd., Osaka, Japan) in CBB buffer (10% acetic acid, 50% methanol, and 40% deionized distilled water (DDW)) for 30 min, and de-stained with CBB buffer overnight.

### Picrosirius red staining

MC cells were seeded onto 15 mm-diameter cover glass (Matsunami Glass Ind., Ltd., Osaka, Japan) placed in the 6-well culture plates. After 2 weeks of culture in the matrix formation medium, the matrix was fixed with 4% formaldehyde, incubated with Picrosirius Red solution (0.1% direct red (Sigma-Aldrich) in saturated picric acid in water (Sigma-Aldrich)) for 30 min, and washed thoroughly with DDW. Samples were analyzed using a bright-field/polarized light microscope (BX53, Olympus, Tokyo, Japan). Alignment of collagen was visually assessed on Picrosirius Red stained samples under polarized light using three sections/group [26]. To semi-quantitatively assess the maturation of collagen matrix, digital images were captured by CCD camera (DP-72, Olympus) and the intensity of RGB signal in the obtained images was quantified using ImageJ software (NIH, Bethesda, MD, USA). Red and green signals representing mature and immature collagenous matrix, respectively, were separately analyzed. Each image was binarized using a fixed threshold, and a positive pixel ratio was calculated [27].

### Collagen cross-link analysis

For cultured matrices, cells/matrices were scraped, thoroughly washed with cold PBS and cold DDW, and lyophilized. Pulverized bone samples from mouse humeri were washed several times with PBS and cold DDW, centrifuged at 4,000 × g for 30 min, and lyophilized. Bone powder was then demineralized with 0.5 M ethylenediaminetetraacetic acid (EDTA), pH 7.5, for 2 weeks with several changes of EDTA solution at 4,000 × g. The EDTA-insoluble residue was thoroughly washed with cold DDW by repeated centrifugation at 4,000 × g and lyophilized. Dried cell/matrix and bone samples (approximately 2.0 mg each) were suspended in buffer containing 0.15 M N-trismethyl-2-aminoethanesulfonic acid, and 0.05 M Tris-HCl, pH 7.4, reduced with standardized NaB^3^H_4_. The specific activity of the NaB^3^H_4_ was determined as described previously [28, 29]. The reduced samples were washed with cold DDW several times by repeated centrifugation at 4,000 × g and lyophilized. Reduced collagen was hydrolyzed with 6 N HCl and subjected to amino acid analysis as described previously [30]. The collagen content per protein was evaluated by the level of hydroxyproline (Hyp) per 1,000 total amino acids. The level of hydroxylysine (Hyl) in a collagen molecule was calculated based on the value of 300 residues of Hyp per collagen molecule. Reduced/hydrolyzed collagen was subjected to cross-link analysis as described previously [30]. Upon reduction, dehydrodihydroxylysinonorleucine (dehydro-DHLNL)/its ketoamine and dehydrohydroxylysinonorleucine (dehydro-HLNL)/its ketoamine are reduced to stable secondary amines, DHLNL and HLNL. The reducible cross-links were analyzed as their reduced forms (i.e. DHLNL and HLNL). Hereafter, the terms DHLNL and HLNL will be used for both the unreduced and reduced forms. The levels of immature reducible (DHLNL and HLNL) and mature non-reducible cross-links (pyridinoline and deoxypyridinoline) were quantified as mol/mol of collagen.

### LOX activity assay

The prepared matrices were homogenized in the 200 μL of extraction buffer (6 M Urea, 10 mM Tris-HCl pH 7.4) overnight at 4°C and supernatants were collected by centrifugation at 20,000 × g for 5 min. LOX activity was determined by commercially available lysyl oxidase activity assay kit (Abcam, Cambridge, UK) by following the manufacturer’s instructions. After incubation for 30 min at 37°C, fluorescence signal was measured by a GloMax Discover System (Promega Corp., Madison, WI, USA) at excitation/emission wavelengths of 520/580-640 nm. Relative LOX activity was calculated as DOC untreated control matrices as a reference.

### Cell culture on prepared collagen matrices

The prepared matrices were further treated with 0.5% of deoxycholate (DOC) for 10 min at 4°C and washed with PBS three times to remove cellular components.

MC cells were seeded onto the low cross-linked matrices in 35-mm dishes at a density of 2 × 10^5^ cells/dish. After 2 days, the medium was replaced with the differentiation medium (alpha-MEM supplemented with 10% FBS, 1% penicillin/streptomycin, 50 μg/mL of ascorbic acid, and 2 mM of beta-glycerophosphate (Sigma-Aldrich)) and cultured for 3 and 7 days.

The RAW264.7 monocyte/macrophage cell line was obtained from ATCC and maintained in in Dulbecco’s Modified Eagle Medium (DMEM) (Life Technologies) supplemented with 10% FBS and 1% penicillin/streptomycin. The RAW264.7 cells were seeded onto the prepared matrix in a 35-mm dish, at a density of 1 × 10^5^ cells/dish. After 2 days, the medium was replaced with the osteoclast differentiation medium (DMEM with 10% FBS, 1% penicillin/streptomycin, and 50 ng/mL recombinant soluble receptor activator of nuclear factor-kappa B ligand (sRANKL) (eBioscience, San Diego, CA, USA)).

Bone marrow stromal cells (BMSCs) were obtained from the femurs of 7-week-old C57BL/6J mice (Charles River Laboratories Japan, Inc.). Both sides of femurs were dissected, soft tissues were removed, both ends were cut, and bone marrow was flushed with ice-cold PBS. The collected cells were maintained in alpha-MEM with 10% FBS and 1% penicillin/streptomycin. Only adherent cells were used as BMSCs. The BMSCs were seeded onto the low cross-linked matrices at a density of 2 × 10^5^ cells/dish. Osteoblastic differentiation of BMSCs was induced using differentiation medium (alpha-MEM containing 15% FBS, 1% penicillin/streptomycin, 50 μg/mL ascorbic acid, 2 mM β-glycerophosphate, and 10 nM dexamethasone) for 1 and 2 weeks.

### Cell proliferation assay

Cell proliferation was analyzed using the 3-(4,5-dimethylthiazol-2-yl)-5(3-carboxymethonyphenol)-2-(4-sulfophenyl)-2H-tetrazolium (MTS) assay, as described previously [31]. Absorbance was measured at 490 nm using the iMark microplate absorbance reader (Bio-Rad, Hercules, CA, USA).

### Quantitative real-time polymerase chain reaction (PCR)

Total RNA was isolated from cells cultured on the prepared matrices, using TRIzol reagent (Life Technologies). Taqman Fast Virus 1-Step Master Mix (Life Technologies) was used for real-time PCR analyses. Amplification and detection were performed using an ABI Prism 7000 Sequence detection system (Applied Biosystems, Foster City, CA, USA) in triplicate. PCR probes used in this study were as follows: runt-related transcription factor 2 (*Runx2*)/core-binding factor alpha 1 (*Cbfa1*) (Mm00501582_m1), type I collagen α2 chain (*Col1a2*) (Mm00483888_m1), alkaline phosphatase (*Alpl*) (Mm00475834_m1), Osteocalcin (*Spp1*) (Mm00436767_m1), lysyl oxidase (*Lox*) (Mm00495386_m1), cathepsin K (*Ctsk*) (Mm00484039_m1), nuclear factor of activated T-cells, cytoplasmic 1 (*Nfatc1*) (Mm00479445_m1), dendritic cell-specific transmembrane protein (*DCstamp*) (Mm04209236_m1), and glyceraldehyde-3-phosphate dehydrogenase (*Gapdh*) (Mm99999915_g1). Gene expression levels were normalized to the *Gapdh* levels, and the fold changes were calculated using the 2^-ΔΔCT^ method [32].

### Alkaline phosphatase (ALP) activity assay

After 7 days of culture in the differentiation medium, cells/matrices were washed with PBS, collected with Tris-buffered saline with 1% Tween 20 (TBS-T), and centrifuged at 8,000 × *g* for 30 min. Cellular ALP activity was analyzed using *p*-nitrophenylphosphate (pNPP) (Sigma-Aldrich) as a substrate. The supernatant was incubated in pNPP solution at room temperature for 15 min, and the reaction was stopped with 5 N NaOH. Absorbance at 405 nm was measured using the iMark Microplate Absorbance Reader, while the protein concentration was measured using NanoEX (OPTIMA Inc., Schaumberg, IL, USA). ALP activity was calculated as pNPP/mg of protein [33].

### Tartrate resistant acid phosphate (TRAP) staining

RAW264.7 cells were cultured for 6 days in osteoclast differentiation medium, fixed using 4% formaldehyde, and stained for TRAP with a commercially available kit (Primary Cell Co., Ltd., Sapporo, Japan). TRAP-positive multinucleate cells, containing three or more nuclei, were counted under the microscope in four different fields per dish.

### Adhesion and osteoblastic differentiation of BMSCs

After 1 h of seeding onto the prepared matrices, BMSCs were fixed in 4% formaldehyde, stained by Alexa Fluor 594-conjugated phalloidin (Invitrogen), and enclosed in VECTASHIELD medium with 4',6-diamidino-2-phenylindole (DAPI) (Vector Laboratories, Burlingame, CA, USA).

### Animals

Four-week-old female C57BL/6J mice (CREA Japan Inc., Tokyo, Japan) were used in this study. Thirty-six mice were divided into three groups (n = 12/group): control, low dose BAPN (fed a diet containing 0.1% BAPN), and high dose BAPN (fed a diet containing 0.25% BAPN). After 8 weeks of BAPN administration, the diet was replaced with the control diet in all experimental groups in order to avoid the direct effects of BAPN on the bone cells. Samples were collected at 0, 2, and 4 weeks after switching to the control diet. Femurs were fixed in 4% formaldehyde, and humeri were kept at ‐80°C until further analysis. All animal procedures were reviewed and approved by the Niigata University Ethics Committee (protocol number 255–3).

### Micro-computed tomography (micro-CT)

Bone morphometry was performed in the region of femur epiphysis. Micro-CT images of the distal femur were obtained using 72-kV and 10-μA irradiation from a high-resolution X-ray tomographic system (ELESCAN, Nittetsu Elex, Osaka, Japan). Bone structure analysis was performed using a software tool (TRI/3D-Bon; RATOC System Engineering, Tokyo, Japan), for the reconstruction of a three-dimensional (3D) image with 222 slices, with 21 μm pitch between slices. Regions of interest (ROI) (3.5 × 3 × 3 mm) were trimmed in all samples. For the bone mineral density (BMD) analysis, density calibration curve was calculated using a standardized Phantom Parameter (RATOC System Engineering). For bone morphometry analysis, the numerical quantitative outcome values were obtained by TRI/3D-Bon software.

### Histology and histomorphometry

Following micro-CT analysis, femurs from BAPN-administrated mice were decalcified using 10% EDTA for 3 weeks, and paraffin-embedded histological samples were prepared [27]. Sections were stained with hematoxylin and eosin (H&E), Picrosirius Red, or TRAP staining. Histomorphometric measurements were performed on trabecular bone at the femur epiphysis in accordance with the guidelines of the American Society for Bone and Mineral Research [34]. Number of osteoblasts per bone surface (N.Ob/BS), osteoblast surface per bone surface (Ob.S/BS), number of osteoclasts per bone surface (N.Oc/BS), and osteoclast surface per bone surface (Oc.S/BS) were calculated.

### Statistical analysis

The reproducibility of the cell culture data was confirmed at least by three independent experiments, and measurements were performed in triplicate. For animal experiments, three histological sections were selected from each animal, and cells were counted in each section. For cross-link analysis, three bone samples in each group were collected and analyzed. Data are expressed as mean ± standard deviation. Statistical significance was assessed with analysis of variance (ANOVA), followed by Tukey’s honest significant difference (HSD) test. A p-value < 0.05 was considered significant.

## Results

### Effect of BAPN on matrix formation and type I collagen components

**Fig 1:**
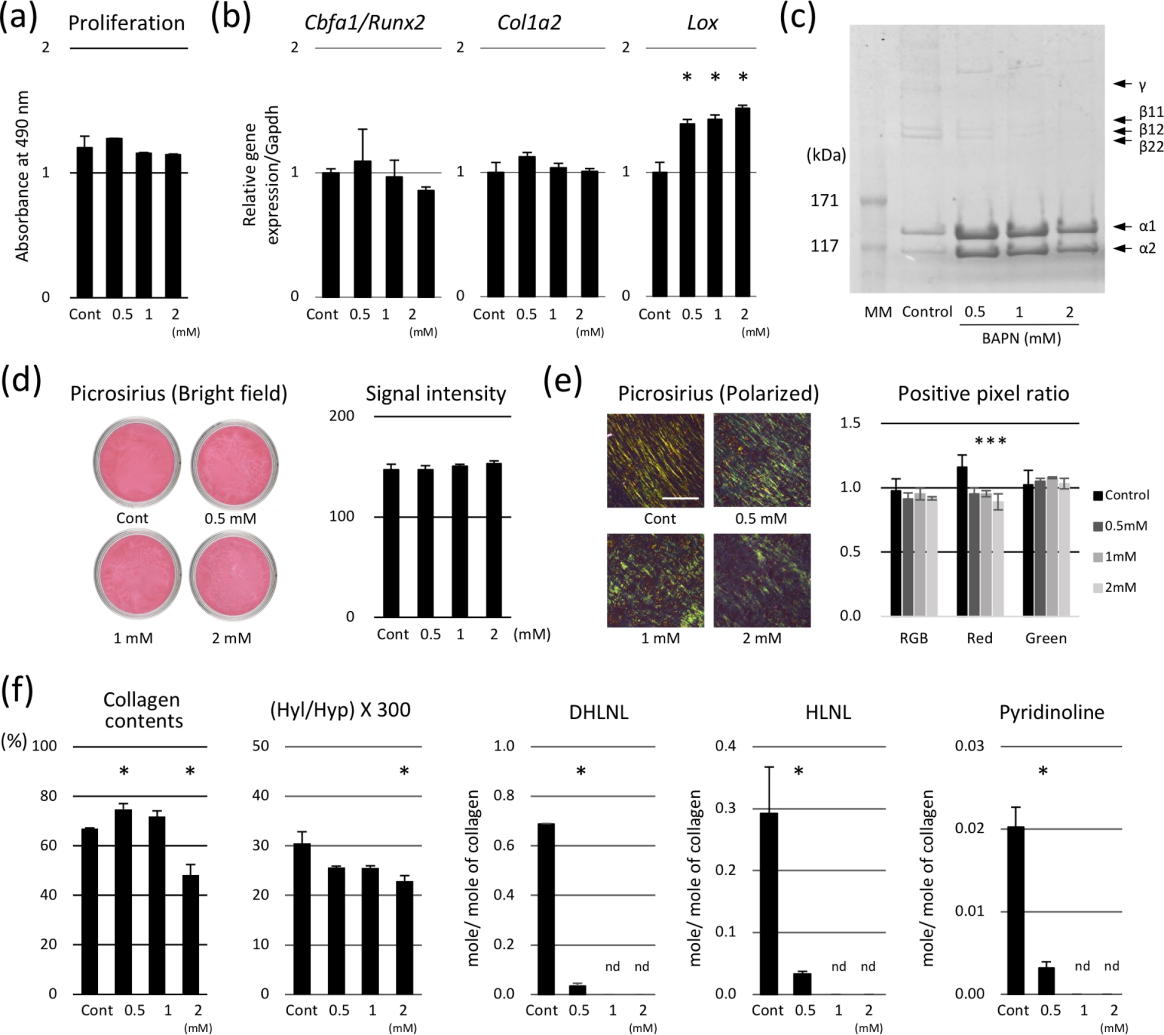
Effect of β-aminopropionitrile (BAPN) on matrix formation and collagen components produced by MC3T3-E1 cells. (a) BAPN treatment did not affect the proliferation of MC cells, as demonstrated by MTS assay. (b) Expression of core-binding factor alpha 1/runt-related transcription factor 2 (*Runx2/Cbfa1*) and type I collagen α2 chain (*Col1a2*) did not change, while the levels of lysyl oxidase (*Lox*) were significantly elevated after BAPN treatment in a dose-dependent manner. (c) Collagen components were analyzed by electrophoresis. In the control, all chains (α, β, and γ) were clearly observable. However, BAPN treatment led to the inhibition of β- and γ-chain formation. Picrosirius Red staining was visualized under bright-field (d) and polarized light (e). Collagen quantity was not affected by BAPN, while the alignment of collagenous fibers and matrix maturation were impaired. Bar: 100 μm. (f) Collagen content was slightly increased following 0.5 mM BAPN treatment, but it decreased after the administration of 2 mM BAPN. Hyl/Hyp × 300 decreased after the treatment with 2 mM BAPN. Divalent DHLNL and HLNL levels decreased with 0.5 mM BAPN treatment and were not detectable following the treatment with 1.0 and 2.0 mM BAPN. Trivalent pyridinoline levels significantly decreased after the application of 0.5 mM BAPN, while they were undetectable following the treatment with 1.0 and 2.0 mM BAPN. *p < 0.05, compared to the control.

Before analyzing the effect of BAPN on cultured matrices, the direct effects of BAPN on MC cells were analyzed. Following the incubation with BAPN for 24 h, cell proliferation did not change (Fig 1A). Expressions of *Runx2/Cbfa1*, an osteoblastic differentiation marker, and *Col1a2*, encoding type I collagen α2 chain, did not change, while the expression of *Lox*, encoding lysyl oxidase, was significantly elevated in a dose-dependent manner (Fig 1B).

Effect of BAPN treatment on the changes in collagen components were analyzed by electrophoresis with CBB staining (Fig 1C). In the control sample, the expression of all collagen components (i.e., α-1, -2, β (dimer), and γ (trimer) chains) was clearly noticeable. However, in the presence of BAPN, the intensities of bands corresponding to β and γ chains decreased, while those of α chains increased.

To analyze the effects of BAPN on MC cell matrix formation, Picrosirius Red staining was performed, and the samples were analyzed using the bright-field and polarized light microscope (Fig 1D and 1E). Bright-field images showed that the collagen content was not affected by BAPN treatment (Fig 1D), while polarized light images showed that the alignment of collagenous fibers and matrix maturation were significantly impaired by this treatment (Fig 1E). Under polarized light, intensity of the red signal, representing mature collagen, significantly decreased, while that of the green signal, representing immature collagen, did not change.

Collagen and its cross-links were further analyzed by HPLC (Fig 1F). Collagen contents and Hyl/Hyp × 300, representing the number of hydroxylated lysine residues per collagen molecule, did not significantly change when ≤ 1.0 mM of BAPN was administered, but they decreased after the application of 2 mM BAPN. The levels of the major collagen cross-links in cultured matrices, DHLNL, HLNL, and pyridinoline significantly decreased following the treatment with 0.5 mM BAPN, and they were undetectable upon treatment with 1.0 and 2.0 mM of BAPN.

### Effect of the matrices with low collagen cross-links on osteoblasts

**Fig 2:**
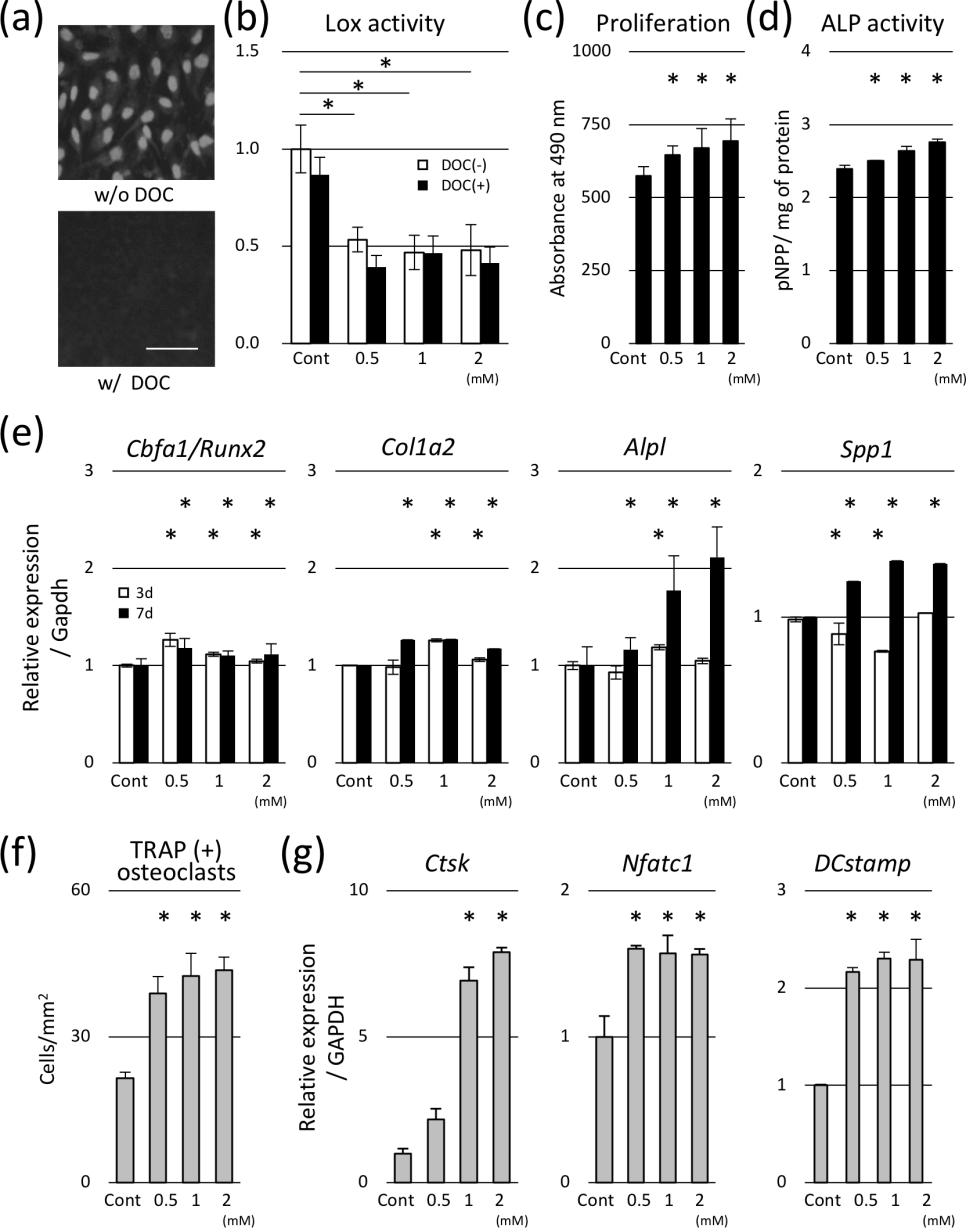
Effect of low cross-linked matrices on osteoblast and osteoclast. (a) The ablation of cellular components by DOC was confirmed by DAPI staining. Bar: 50 μm. (b)The Lox activity of prepared matrices before and after the DOC treatment was analyzed. The Lox activity was significantly decreased in the matrices produced by BAPN-treated cells. DOC treatment did not significantly affect the Lox activity of prepared matrices, regardless of the BAPN concentration. (c) The proliferation of MC cells was significantly increased in the low cross-link density matrices. (d) Alkaline phosphatase (ALP) activity increased in cells seeded on low cross-linked matrices. (e) Gene expression of *Cbfa1/Runx2*, type I collagen α2 chain (*Col1a2*), alkaline phosphatase (*Alpl*), and osteocalcin (*Spp1*) significantly increased in these matrices at 3 and 7 days of culture. (f) Osteoclasts were cultured on differentially cross-linked matrices for 6 days under the differentiation condition. The number of multi-nuclear tartrate-resistant acid phosphatase (TRAP)-positive cells increased in the low cross-link density matrices. (g) Gene expression of cathepsin K (*Ctsk*), nuclear factor of activated T-cells cytoplasmic 1 (*Nfatc1*), and dendritic cell-specific transmembrane protein (*DCstamp*) increased in low cross-link density matrices. *p < 0.05, compared to the control.

To prepare cell-free matrices with altered collagen cross-linking, the cell/matrix layers produced by cells treated with various concentrations of BAPN were further treated with 0.5% of DOC. Cell ablation by DOC was confirmed by DAPI staining, which revealed the absence of nuclei (Fig 2A).

Because gene expression of *Lox* was increased by BAPN treatment (Fig 1B), Lox activity of prepared matrices could be affected. Therefore, Lox activity of prepared matrices was analyzed before and after the DOC treatment (Fig 2B). Lox activity was significantly decreased in the matrices produced by BAPN-treated cells. DOC treatment did not significantly affect the Lox activity of the prepared matrices, regardless of the BAPN concentration.

To analyze the effect of collagen matrices with low cross-linking on the differentiation of osteoblasts, MC cells were seeded onto the cell-free matrices. The proliferation (Fig 2C) and ALP activity (Fig 2D) of MC cells increased in the low cross-linked matrices in a dose-dependent manner. Gene expression of *Cbfa1/Runx2*, *Col1a2*, *Alpl*, *and Spp1* also significantly increased at 3 and 7 days of culture (Fig 2E).

### Effect of low cross-linked matrices on osteoclasts

The differentiation of osteoclasts was analyzed using a monocyte/macrophage cell line, RAW264.7. The number of multi-nuclear TRAP-positive cells significantly increased by the low cross-linked collagen matrices compared to the control (Fig 2F). Expression of *Ctsk*, *Nfatc1*, and *DCstamp*, representing maturation of osteoclasts, also increased when cells were cultured on these matrices (Fig 2G).

### Effect of low cross-linked matrices on the osteoblastic differentiation of BMSCs

**Fig 3:**
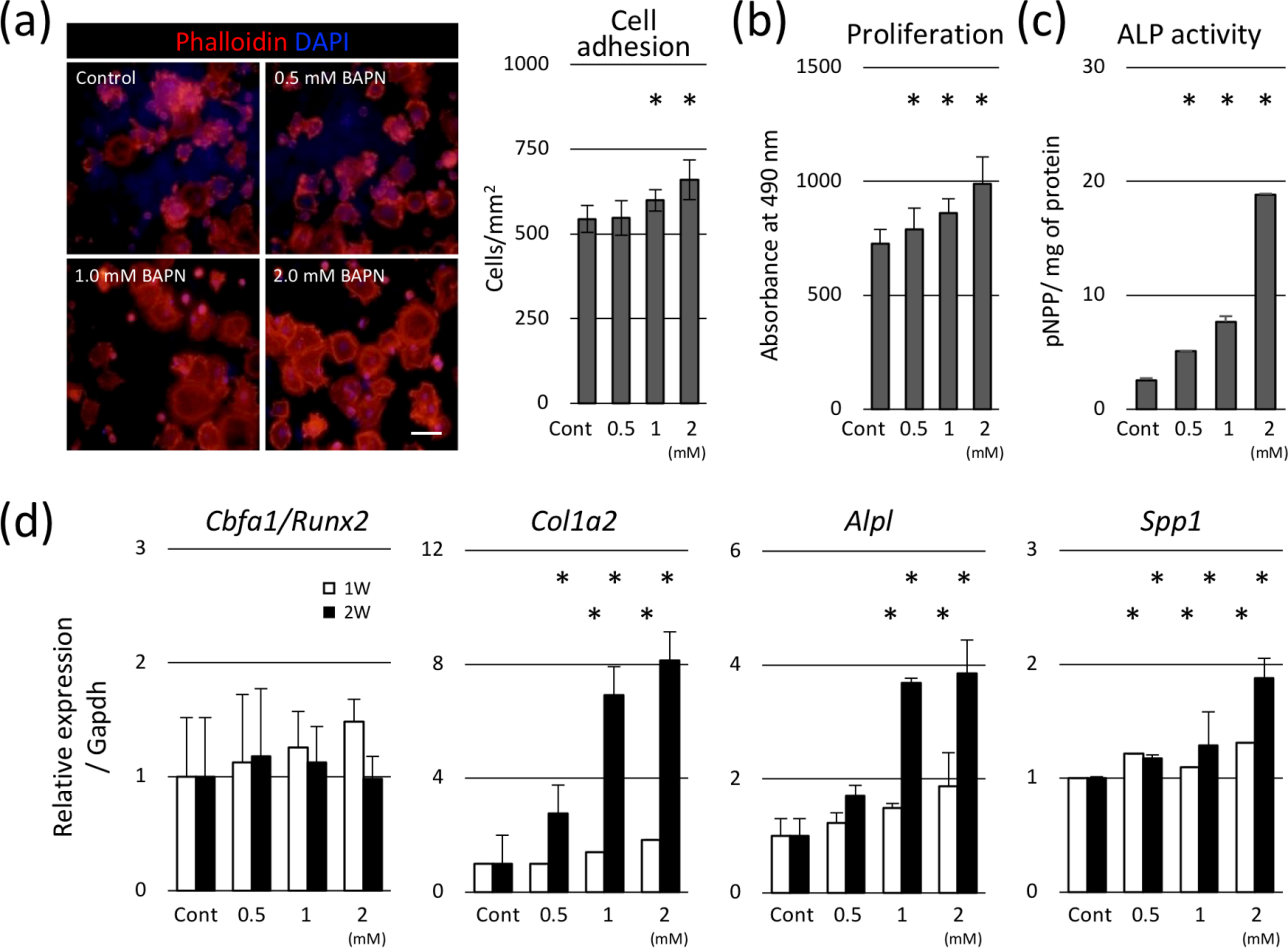
Effect of low cross-linked matrices on osteoblastic differentiation of BMSCs. (a) BMSCs to the prepared matrices was shown. Cell nuclei were stained using DAPI (blue), and the cytoskeleton was stained with phalloidin (red). Initial adhesion of cells cultured on low cross-link density matrix increased. (b) Proliferation of BMSCs was significantly elevated in low cross-linked matrices. (c) ALP activity of BMSCs increased in low cross-link density matrices. (d) Gene expression of *Col1a2*, *Alpl*, and *Spp1* was significantly increased, while that of *Cbfa1/Runx2* did not change in low cross-link density matrices after 1 and 2 weeks. *p < 0.05, compared to the control. Bars: 20 μm.

As a source of osteogenic cells, BMSCs substantially contribute to bone remodeling. Thus, the effect of low cross-linked matrices on the osteoblastic differentiation of BMSCs was also analyzed. Initial adhesion (Fig 3A), proliferation (Fig 3B), and ALP activity (Fig 3C) of BMSCs significantly increased in cells cultured on the low cross-linked matrices. Gene expression of *Col1a2*, *Alpl*, and *Spp1* significantly increased, while that of *Cbfa1/Runx2* did not change in low cross-link density matrices, compared to the control after 1 and 2 weeks (Fig 3D).

### Effects of BAPN-containing diet on body weight, bone volume, and collagen cross-linking in mice

**Fig 4:**
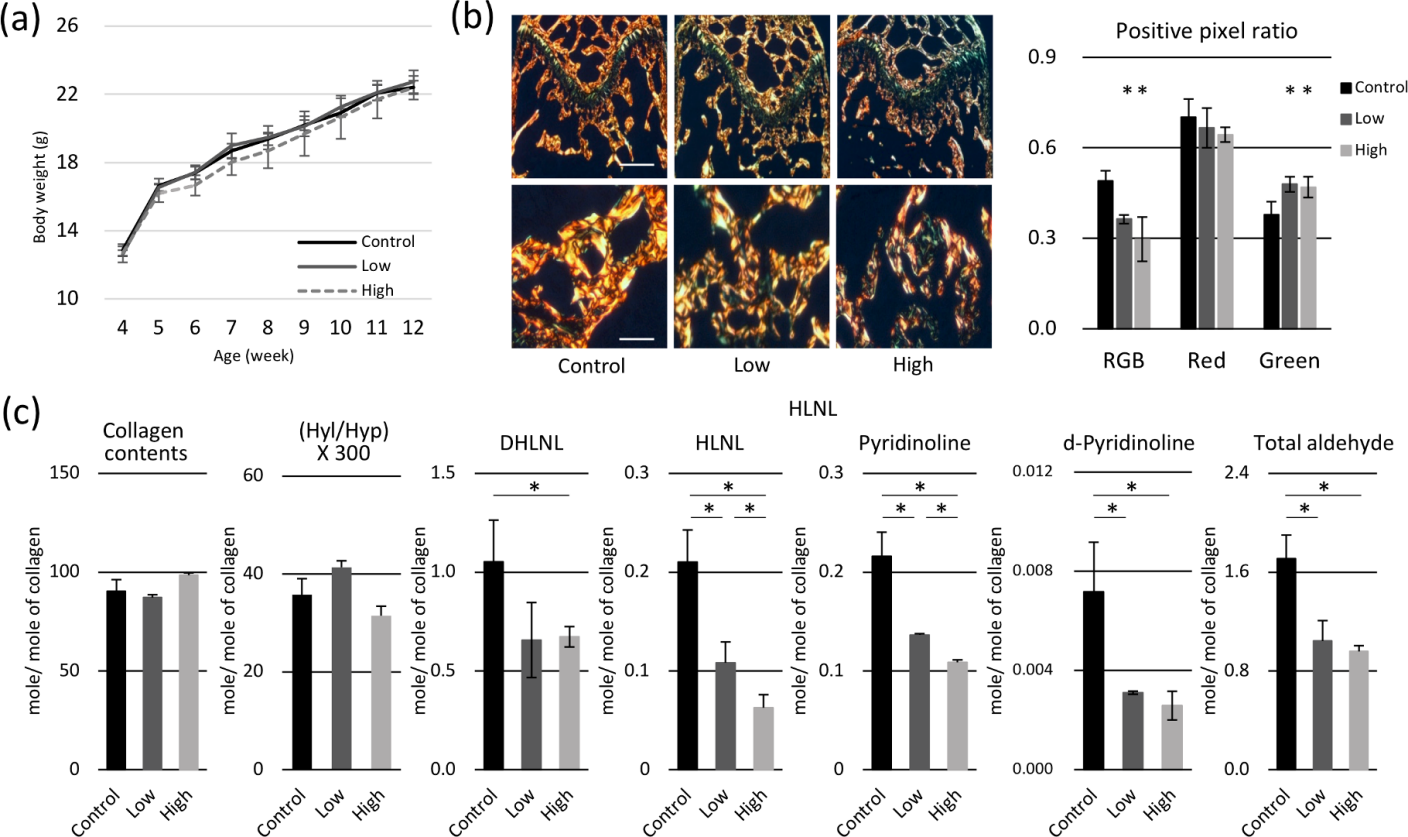
Effects of β-aminopropionitrile (BAPN)-containing diet on body weight, bone volume, and collagen cross-linking in mice. (a) The body weight of mice increased steadily throughout the experimental period, and no differences were observed between groups. (b) Histology of Picrosirius Red-stained samples at 12 weeks of age (after 8-week of the BAPN consumption) is shown. Under polarized light, positive pixel ratio of immature matrix (green) increased with BAPN administration, while that of mature matrix (red) did not change Bar: 200 μm. (c) Collagen Cross-links were analyzed at 12 weeks of age (after 8-week of the BAPN consumption). Collagen content and hydroxylysine (Hyl)/hydroxyproline (Hyp) × 300 were not affected by BAPN, but dehydrodihydroxylysinonorleucine (DHLNL) and dehydrohydroxylysinonorleucine (HLNL) levels decreased in a dose-dependent manner. Pyridinoline, deoxypyridinoline, and total aldehyde contents were significantly reduced in mice fed with BAPN-containing diet. *p < 0.05, compared to the control.

In order to confirm the above findings *in vivo*, 4-week-old mice were fed a diet without or with two doses of BAPN (Low and High) for 8 weeks. Mouse body weight increased steadily throughout the experimental period, and no differences were observed among control and experimental groups (Fig 4A). To examine the effect of BAPN administration on bones, femur epiphysis was characterized by micro-CT. Among the parameters analyzed, tissue and bone volumes tended to increase in mice that received BAPN; however, these changes were subtle, and no significant differences were detected (Table 1). Additionally, histological sections were stained with Picrosirius Red and observed under polarized light (Fig 4B). Positive pixel ratio of immature matrix (green) increased with BAPN administration, while that of mature matrix (red) did not change. Collagen content and cross-links were further quantitatively analyzed by HPLC (Fig 4C). Collagen contents and the hydroxylation of lysine residues were not significantly affected by BAPN, but the levels of divalent (DHLNL and HLNL) and trivalent cross-links (pyridinoline and deoxypyridinoline) significantly decreased in a dose-dependent manner. Total aldehyde content involved in these cross-links significantly decreased with the BAPN treatment.

**Table 1:** Morphometric analysis of femur.

	Control	Low-dose BAPN		High-dose BAPN	
BMD (mg/cm^3^)	337.37 ± 20.96	357.79 ± 18.91		345.87 ± 33.96	
BMC (mg)	0.11 ± 0.05	0.12 ± 0.04		0.15 ± 0.05	
TV (mm^3^)	4.46 ± 0.29	4.78 ± 0.20	*	4.96 ± 0.24	*
BV (mm^3^)	0.38 ± 0.08	0.44 ± 0.05		0.46 ± 0.05	
BS (mm^2^)	19.45 ± 2.83	21.11 ± 1.42		22.74 ± 2.04	*
BS/BV (1/mm)	51.09 ± 3.06	48.51 ± 2.72		49.64 ± 1.91	
BS/TV (1/mm)	4.36 ± 0.61	4.41 ± 0.24		4.59 ± 0.32	
BV/TV (%)	8.62 ± 1.76	9.14 ± 0.96		9.26 ± 0.88	
Tb.Th (μm)	39.27 ± 2.43	41.34 ± 2.38		40.34 ± 1.53	
Tb.N (1/mm)	2.18 ± 0.30	2.21 ± 0.12		2.29 ± 0.16	
Tb.Sp (μm)	426.35 ± 59.32	413.11 ± 26.59		397.47 ± 32.48	
Tb.Spac (μm)	465.62 ± 57.15	454.45 ± 24.93		437.81 ± 31.58	

BMD, bone mineral density; BMC, bone mineral content; TV, tissue volume; BV, bone volume; BS, bone surface; BS/TV, specific surface of bone; BV/TV, bone volume fraction; Tb.Th, trabecular thickness; Tb.N, trabecular number; Tb.Sp, trabecular separation; Tb.Spac, trabecular spacing

*p < 0.05.

### Histology

**Fig 5:**
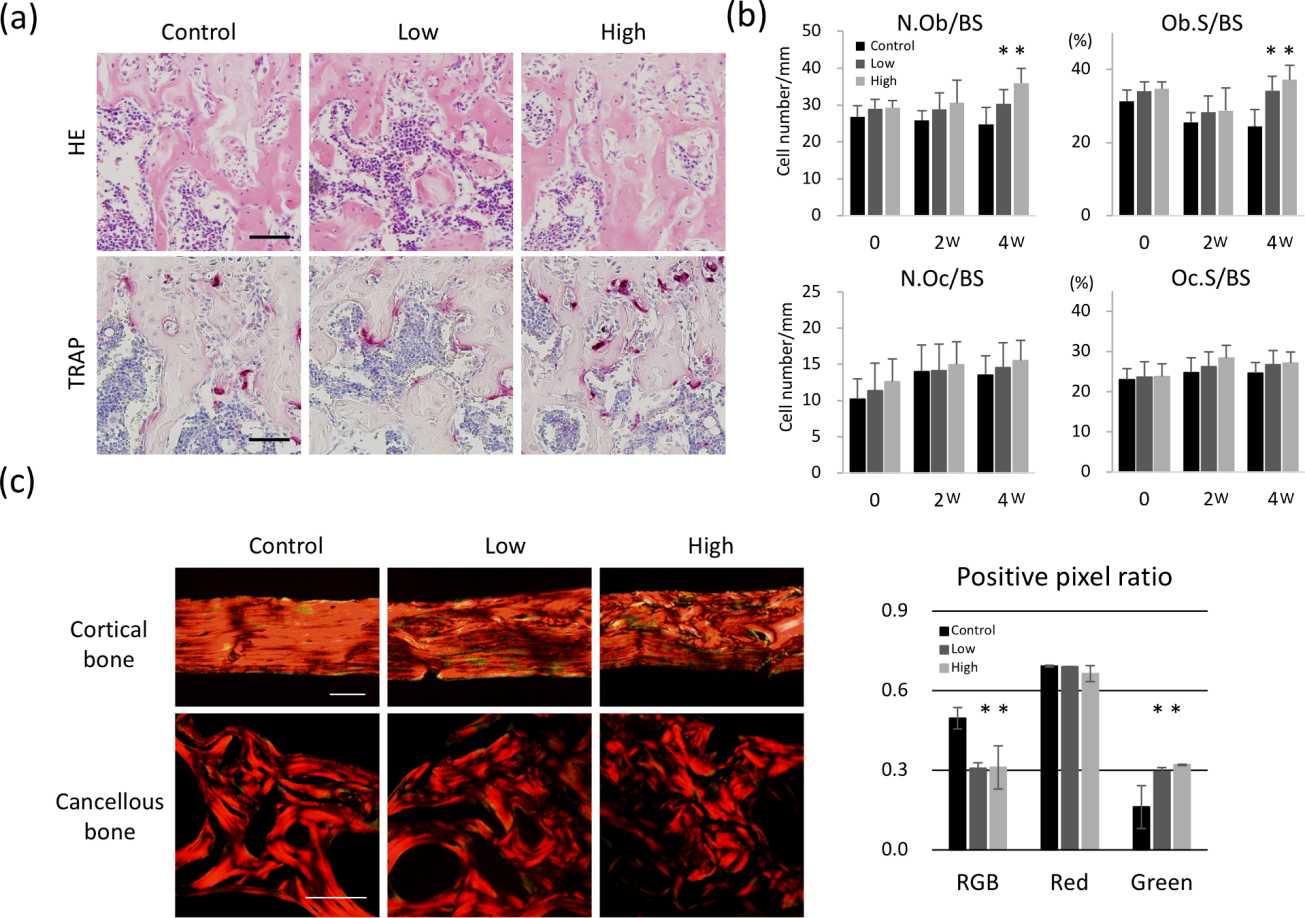
Histological and histomorphometric analysis. (a) Hematoxylin and eosin, and tartrate-resistant acid phosphatase (TRAP)-stained histological sections of the distal femur epiphysis at 0 week (after 8-week of the BAPN consumption). (b) No differences of osteoblast and osteoclasts activities were observed at 0 and 2 weeks after replacing to the control diet. After 4 weeks, number of osteoblasts per bone surface (N.Ob/BS) and osteoblast surface per bone surface (Ob.S/BS), representing osteoblast activity, significantly increased, while number of osteoclasts per bone surface (N.Oc/BS) and osteoclast surface per bone surface (Oc.S/BS), representing osteoclast activity did not change. *p < 0.05, compared to the control. Bar: 50 μm. (c) Picrosirius red-stained samples at 16 weeks of age (after 8-week of the BAPN consumption followed by 4-week of control diet) are analyzed under polarized light. After 4-week of control diet, immature/irregular collagen matrix still detected both in cortical and cancellous bone. Bar: 50 μm. Quantitative data also confirmed that the immature collagens, detected in green, retained high value after 4-week of control diet in cancellous bone.

The effects of BAPN administration on the osteoblasts and osteoclasts in bone were also analyzed by H&E and TRAP staining (Fig 5A). Consistent with the results obtained using bone histomorphometry and micro-CT, BAPN treatment for 8 weeks did not affect osteoblast and osteoclast activities. During the period of BAPN administration, cells could be affected not only by the low cross-linked matrices produced by themselves or neighboring cells, but also directly by BAPN. In order to minimize the direct effect of BAPN to the cells, the diet of all groups was replaced with the control diet and analyzed 2 and 4 weeks later. Four weeks after switching to the control diet, osteoblast activities (N.Ob/BS and Ob.S/BS) significantly increased in both low- and high-dose BAPN groups, while osteoclast activities (N.Oc/BS and Oc.s/BS) did not change (Fig 5B). In addition, to confirm the remaining of the BAPN-affected low cross-linked bone after 4-week of normal diet, histology samples were stained by picrosirius red and analyzed under polarized light (Fig 5C). After 4-week of control diet, immature/irregular collagen matrix still detected both in cortical and cancellous bone. Quantitative data also showed that the immature collagens still exist after 4-week of control diet in cancellous bone, confirming that the surrounding bone cells are still under the influence of low cross-linked matrices.

## Discussion

Collagen cross-linking is one of the major determinants of bone quality, which is associated with bone mechanical properties. Therefore, it has been considered as an important predictor of bone fracture risks. Although the biochemical and biophysical status of type I collagen affects cell behavior, the significance of collagen cross-linking for the activity of bone cells and subsequent tissue remodeling is not well understood. Therefore, in this study, we investigated the potential contribution of collagen cross-links in bone cellular activities, by employing *in vitro* and *in vivo* approaches. To generate the decellularized matrices with low collagen cross-links, we used a potent lathyrogen, BAPN, which can disrupt collagen cross-linking by irreversibly inhibiting lysyl oxidase activity. BAPN has been widely used for the induction of abnormal cross-linking in matrices both *in vitro* [23, 35] and *in vivo* [27, 35–39]. In the present study, we showed that, up to certain concentration, BAPN efficiently suppressed the formation of collagen cross-links without affecting collagen production. The osteoblasts, osteoclasts, and BMSCs that were cultured on collagen matrices having low cross-links differentiated more rapidly than those on normal collagen matrices. To the best of our knowledge, this is the first evidence that altered collagen cross-linking affects the differentiation of osteoclasts. These results suggest that osteoblast- and osteoclast-mediated bone tissue remodeling is affected by the altered collagen cross-linking of bone matrix.

Previous studies have tried to demonstrate the significance of collagen cross-linking for the differentiation of osteoblasts by using BAPN [23, 25]. Although these studies concluded that collagen cross-links influenced the osteoblastic differentiation, they analyzed the differentiation of osteoblasts in the presence of BAPN. Under this condition, BAPN affects not only collagen cross-linking but also the differentiation of osteoblasts [23, 24]. Thus, it is not clear if the effect on cells was due to altered collagen cross-linking or BAPN itself. In the present study, low cross-linked collagen matrices were prepared by using BAPN, cellular components were washed out by DOC, and the effect of the low cross-linked matrices was analyzed with newly prepared cells under BAPN-free condition. This protocol enabled the analysis of the effect of altered collagen cross-linking to the differentiation of bone cells in a reliable manner. The same approach was employed in animal studies, in which BAPN containing diet was fed for 8 weeks to the mice and switched to control diet for 4 weeks to minimize the direct effect of BAPN administration.

In the present study, we found that BAPN treatment significantly increased the gene expression of *Lox* in MC cells after 24 hours (Fig 1B), which may affect the collagen cross-linking of the matrices. Therefore, we further examined the Lox activity in the prepared matrices. The data confirmed that increased gene expression of *Lox* was not associated with the Lox activity in the prepared matrices (Fig 2B). Furthermore, cross-link analysis clearly showed that BAPN inhibited the collagen cross-links in a dose dependent manner (Fig 1F). Therefore, the increased *Lox* expression and subsequent production of Lox protein was most likely offset by BAPN added in the culture. Turecek et al. reported contradictory results, i.e. 0.4 mM of BAPN treatment of MC cells for 1 week significantly decreased the *Lox* gene expression [23]. The reason for the discrepancy is not clear but it could be due to the differences in the BAPN concentration used and/or culture conditions such as the length of culture time.

In a previous study, 4 weeks of daily intraperitoneal injection of BAPN induced 45% reduction of pyridinoline cross-link content, which resulted in 26% reduction in the bending strength [36]. A recent study has also reported that 3 weeks of dose-controlled intraperitoneal administration of BAPN was able to inhibit collagen cross-linking and to reduce bone fracture toughness, while no significant changes were observed in bone mineral density [35]. The changes in DHLNL and HLNL cross-links were not significant, while pyridinoline was reduced by only 10%. The results obtained in this study showed that 8 weeks of oral administration of BAPN-containing diet successfully inhibited both divalent (DHLNL and HLNL) and trivalent (pyridinoline and d-pyridinoline) cross-link formation in bone, which led to the approximately 40% reduction in total aldehyde involved in these cross-links. Our results also indicate that at least 40% of bone matrix was remodeled during 4–12 weeks of age and BAPN-affected collagen was incorporated in the matrix. The 40% reduction of LOX-generated aldehydes in bone collagen is a significant change and Picrosirius Red staining confirmed that collagen alignment and maturation were severely impaired.

There were inconsistencies in the relative amounts of immature vs mature collagen matrices seen in picrosirius red staining and osteoclast function between in vitro and in vivo experiments. The inconsistencies could be due to the difference in experimental conditions. First, the doses of BAPN that affected cells in vitro and in vivo could be different. It is technically challenging to estimate the actual concentration of BAPN when it reached the bone cells in vivo. Second, not all collagen in bone was affected by BAPN in vivo because of the presence of collagen prior to BAPN treatment. On the contrary, in an in vitro experiment, as there are no pre-existing collagen, all collagen was synthesized and deposited by the cells affected by the dose of BAPN used. Consequently, relative amounts of immature vs mature collagen matrices are different between in vitro and in vivo experiment. These factors could also explain the difference in the effects on osteoclasts between in vitro and in vivo. In previous studies, an increase in the number of osteoclasts was observed following the administration of BAPN at the alveolar bone surface in mice [40] and rats [37]. It has been reported that the turnover rate of alveolar bone is faster than that of femur [41]. Thus, a low cross-linked collagen matrix may accumulate more in alveolar bone than femur. The abundance of immature cross-links in mandibular bone collagen suggests that alveolar bone collagen is relatively immature and, thus, more susceptible to degradation and turnover [42]. In fact, the rate of postmenopausal bone loss in rats differs depending on the site and type of bone [43]. Although we could not detect the difference in osteoclast numbers in the femur cancellous bone of BAPN-administrated rats, it is possible that the effect of BAPN on bone matrix could be different depending on the location and/or type of bone.

As discussed above, the effect of BAPN on bone collagen cannot simply be compared only by dose, because various factors such as administration period, administration route, animal age and species, influence the collagen synthesis by BAPN-affected cells. But when we use the extent of collagen cross-linking as an indicator, oral administration of low dose (0.1%) BAPN containing diet to the 4 week-old mice for 8 weeks diminished pyridinoline by 50%, and d-pyridinoline by 64% in bone collagen (Fig 4C). A previous study reported that the treatment of 5 week-old mice with a high BAPN dose (500 mg/kg of body weight) through intraperitoneal injection for 3 weeks resulted in only 10% reduction of total pyridinium cross-links [6]. This indicates that, compared to the previous injection model, our diet model inhibited collagen cross-linking more effectively in bone.

Collagen cross-linking is one of the determinants of matrix stiffness in many connective tissues including bone [44]. Previous studies showed that osteoblastic differentiation was accelerated when cells were grown on stiff substrate [20, 45, 46]. However, in this study, low cross-linked collagen matrices, which are presumably less stiff, accelerated adhesion, proliferation and differentiation of osteoblasts. The reason for these effects are not clear at this point, but possibly low cross-linked collagen may make its cell-binding sites more accessible for cells to exert these effects.

Collagens are the most commonly used scaffold for the bone tissue engineering, and BMSCs are often applied to the scaffold owing to their high proliferative and multipotent properties [47]. Collagen scaffolds need to be artificially cross-linked to hold the shape and to anchor the cells. In addition, defining proper mechanical properties and molecular cues is essential for the behaviors of the osteoblast precursors, including BMSCs. Our results clearly demonstrated that low cross-linked collagen matrices accelerated the adhesion, proliferation, and osteoblastic differentiation of BMSCs. Interestingly, our results showed that low crosslinked matrices increased the gene expression of *Cbfa1/Runx2*, an early regulator of osteoblastic differentiation, in osteoblasts (MC), but not in BMSCs. This may indicate that low cross-linked matrices accelerate the osteoblastic differentiation rather than initiating osteoblastogenesis. Subtle control of the collagen cross-linking in the artificial scaffold may optimize the efficacy of regenerative therapy by directing the expansion and osteoblastic differentiation of BMSCs [48].

Collagen cross-linking is crucial to stabilize collagen molecules in the fibrils and it has long been recognized as one of the major determinants of bone mechanical properties. However, its impact on cell behavior is not well understood. Our results clearly demonstrate that the extent of collagen cross-linking of matrix affects the differentiation of bone cells, indicating its role in the regulation of cell behavior and, possibly, bone tissue turnover. Recent studies have revealed that collagen cross-linking was associated with the tumor cell invasion and metastasis [49, 50], strongly supporting its significant role in the regulation of cell behaviors. Thus, characterization of collagen cross-linking in bone may provide some insight into the rate of bone turnover, which is another important determinant of bone quality [51, 52]. Currently, the gold standard of collagen cross-link quantification depends on the HPLC-based biochemical analysis, thus, invasive. The development of non-invasive collagen cross-linking analysis may be beneficial to obtain insight into bone mechanical property and cellular activities in clinical settings.

## Abbreviations

alpha-MEM: alpha-minimum essential medium
BAPN: β-aminopropionitrile
BMD: bone mineral density
BMSC: bone marrow stromal cell
DHLNL: dehydrodihydroxylysinonorleucine
HLNL: dehydrohydroxylysinonorleucine
Hyl: hydroxylysine
Hyp: hydroxyproline
MC: MC3T3-E1 cell
MSC: Mesenchymal stem cell
pNPP: *p*-nitrophenylphosphate
ROI: region of interest
TRAP: tartrate resistant acid phosphate
